# NAD(H) phosphates mediate tetramer assembly of human C-terminal binding protein (CtBP)

**DOI:** 10.1016/j.jbc.2021.100351

**Published:** 2021-01-30

**Authors:** Jeffry C. Nichols, Celia A. Schiffer, William E. Royer

**Affiliations:** 1 Department of Biochemistry and Molecular Pharmacology, University of Massachusetts Medical School, Worcester, Massachusetts, USA; 2 Chemistry Department, Worcester State University, Worcester, Massachusetts, USA

**Keywords:** CtBP, transcription coregulator, tetrameric assembly, NAD(H), MALS, cancer target, dehydrogenase, structural biology, crystallography, CtBP, C-terminal binding protein, D2-HDH, D-isomer-specific 2-hydroxyacid dehydrogenase, MALS, multiangle light scattering, SEC, size-exclusion column, TCEP, tris(2-carboxyethyl) phosphine

## Abstract

C-terminal binding proteins (CtBPs) are cotranscriptional factors that play key roles in cell fate. We have previously shown that NAD(H) promotes the assembly of similar tetramers from either human CtBP1 and CtBP2 and that CtBP2 tetramer destabilizing mutants are defective for oncogenic activity. To assist structure-based design efforts for compounds that disrupt CtBP tetramerization, it is essential to understand how NAD(H) triggers tetramer assembly. Here, we investigate the moieties within NAD(H) that are responsible for triggering tetramer formation. Using multiangle light scattering (MALS), we show that ADP is able to promote tetramer formation of both CtBP1 and CtBP2, whereas AMP promotes tetramer assembly of CtBP1, but not CtBP2. Other NAD(H) moieties that lack the adenosine phosphate, including adenosine and those incorporating nicotinamide, all fail to promote tetramer assembly. Our crystal structures of CtBP1 with AMP reveal participation of the adenosine phosphate in the tetrameric interface, pinpointing its central role in NAD(H)-linked assembly. CtBP1 and CtBP2 have overlapping but unique roles, suggesting that a detailed understanding of their unique structural properties might have utility in the design of paralog-specific inhibitors. We investigated the different responses to AMP through a series of site-directed mutants at 13 positions. These mutations reveal a central role for a hinge segment, which we term the 120s hinge that connects the substrate with coenzyme-binding domains and influences nucleotide binding and tetramer assembly. Our results provide insight into suitable pockets to explore in structure-based drug design to interfere with cotranscriptional activity of CtBP in cancer.

C-terminal binding proteins (CtBPs) are transcriptional coregulators that were first identified through interactions with the C-terminal region of the adenovirus E1A oncoprotein that modulate E1A transforming activities (1, 2). CtBP1 and CtBP2 regulate cellular processes through binding transcription factors and recruiting chromatin remodeling enzymes such as histone deacetylases, methyl transferases, and demethylases to targeted promoters (3, 4, 5). CtBP cotranscriptional function has been shown to be important in normal embryogenesis in model organisms (6, 7, 8, 9). Knockout experiments in mice reveal distinct roles for CtBP1 and CtBP2 in development, with the loss of CtBP2 embryonically lethal, whereas CtBP1-null mice are small but the majority survive (6).

Numerous lines of evidence implicate human CtBP in cancer progression. Both CtBP paralogues are global repressors of the epithelial phenotype and of apoptotic pathways (3) by acting as corepressors of genes including tumor suppressive proapoptotic factors (*Bik*, *Noxa*), cytoskeletal/cell adhesion molecules (*keratin-8*, *E-cadherin*), and cell-cycle inhibitors (4, 10). CtBP has also been found to be a coactivator of growth and metastasis-related genes (*Tiam1*, *MDR1*, certain Wnt target genes), which facilitate the epithelial-to-mesenchymal transition (EMT) (11, 12, 13, 14). Consistent with a role in repression of apoptotic pathways and activation of growth and metastasis, CtBP is upregulated in a number of cancer tissues including colorectal cancer (15), melanoma (16), metastatic prostate cancer (17), esophageal squamous cell carcinoma (18), ovarian cancer (19), and breast cancer (20, 21). Strikingly, elevated levels of CtBP in tumor tissue have been correlated with poorer survival in breast cancer (22), ovarian cancer (19), gastric carcinoma (23), and hepatocellular carcinoma (24). Recent results add to evidence of a link between CtBP and cancer progression by showing increased survival in mice models for colon cancer (*APC*^*min/+*^) (25) and pancreatic adenocarcinoma (CKP) (26) when CtBP2 levels are lowered by *CtBP2*^*+/-*^ heterozygosity.

A unique feature of CtBP is the incorporation of a D-isomer-specific 2-hydroxyacid dehydrogenase (D2-HDH) domain, which reduces or oxidizes substrates using the coenzyme NAD(P)^+^/NAD(P)H (27, 28). While evidence indicates that catalytic activity is not required for some CtBP activities (10, 29), mutant studies suggest that catalytic activity of CtBP can be important in *Drosophila melanogaster* development (30). Regardless of the cellular relevance of this catalytic activity, the deep substrate and coenzyme cavities suggest that CtBP may provide a particularly favorable target for the development of small-molecule inhibitors in cancer.

Oligomerization of transcriptional factors is an important mechanism for regulation of gene expression (31, 32). In the case of CtBP, substantial evidence exists that oligomerization is linked with NAD(H) binding (5, 29, 33, 34, 35, 36), and dimer-destabilizing mutants inhibit transcriptional function (37, 38, 39, 40). Although assembly of CtBP has primarily been considered in terms of dimers, there is accumulating evidence that NAD(H) binding triggers the assembly of two CtBP dimers to form tetrameric dimers of dimers (33, 34, 35, 41, 42). Moreover, we have recently shown that tetramer-destabilizing mutants of CtBP2 are defective for oncogenic activity (42). Here we explore the moieties within NAD(H) that are responsible for promoting tetrameric assembly. Our multiangle light scattering (MALS) data show that ADP promotes tetrameric assembly in CtBP1 and CtBP2, whereas AMP promotes tetrameric assembly for only CtBP1. By mutagenesis, we pinpoint the basis for different AMP responses between the two paralogs to a short segment that both connects the two subdomains and participates in the tetrameric interface. Moieties of NAD(H) lacking the adenosine phosphate are unable to promote tetrameric assembly. To understand the structural basis for AMP-linked assembly of CtBP1, we determined the crystal structure of CtBP1 with AMP, which reveals a direct interaction involving the adenosine phosphate in tetramer formation. Thus, this phosphate is critical for the response of CtBP to binding of NAD(H).

## Results

### Multiangle light scattering reveals the extent of CtBP1 and CtBP2 tetrameric assembly in the presence of NAD(H) moieties

To ascertain the basis for NAD(H)-linked tetramer formation, the level of assembly of CtBP1 and CtBP2 in the presence of NAD(H) moieties (Fig. S1) was investigated by MALS of samples eluting from a size-exclusion column (SEC). (NAD(H) refers to either NADH or NAD^+^.) Previously we demonstrated, also with SEC-MALS, the linkage between NAD(H) and tetrameric assembly of CtBP1 and CtBP2 (41).

**Figure 1:**
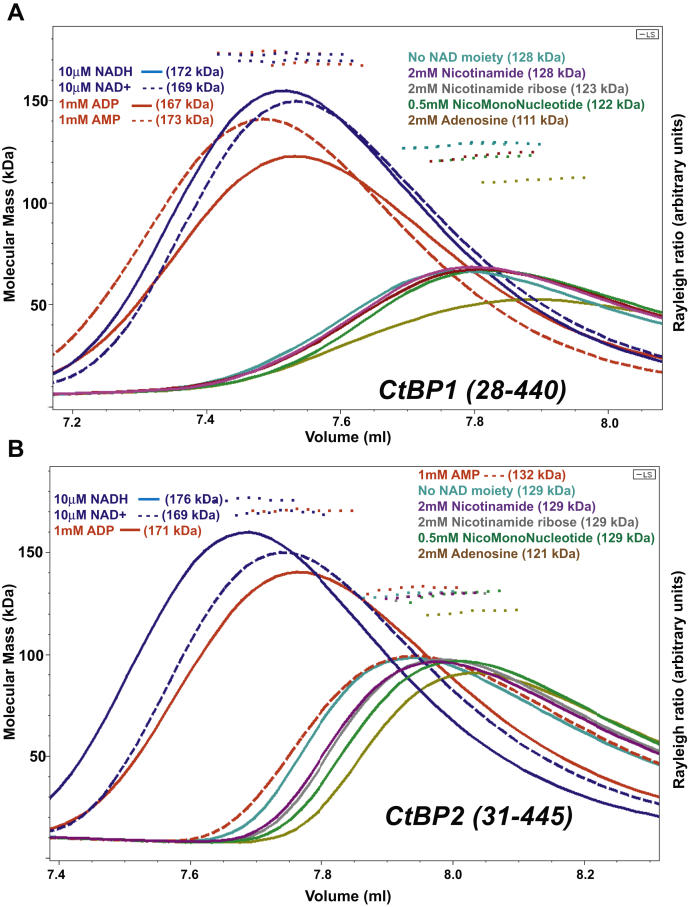
**SEC trace and MALS molecular masses for *A*, CtBP1 (28–440) and *B*, CtBP2 (31–445) in the presence of NAD(H) moieties.** The lines (*continuous* or *dashed*) show the light-scattering Rayleigh ratio (arbitrary units) for protein elution from the SEC column, and the small squares show the MALS molecular mass measurements across the elution peaks. The plots reveal that tetramer formation is promoted by NADH, NAD^+^, and ADP in both CtBP1 and CtBP2, but by AMP only in CtBP1 at the concentrations shown. (The elution peak concentrations ranged from 1.6 μM to 3.1 μM for CtBP1 and 1.6 μM to 2.0 μM for CtBP2. Tabulation of these data, along with replicate experiments, is provided in Table S1.)

Results of our SEC-MALS experiments on CtBP1 (28–440) (herein referred to as CtBP1) and CtBP2 (31–445) (herein CtBP2) are shown in Figure 1. Consistent with our previous work (41), CtBP1 is primarily tetrameric in the presence of 10 μM NADH and 10 μM NAD^+^, with weight average mass (M_w_) of 172 and 169 kDa, respectively. Additionally, 1 mM ADP and 1 mM AMP also exhibit early elution and high molecular masses (M_w_ = 167–173 kDa). CtBP1 in the presence of the other tested compounds elutes later and shows molecular mass values of 111–128 kDa, similar to CtBP1 in the absence of any NAD moiety (128 kDa). The observed molecular masses indicate heterogeneous mixtures, primarily of 94 Da dimers and 188 kDa tetramers. Given that light scattering provides weight average molecular mass estimates (M_w_) (43), one can calculate approximate molar fractions of dimers and tetramers, assuming that those are the only species contributing to the light scattering (see Experimental procedures). Based on these assumptions, CtBP1 in NADH, NAD^+^, AMP, and ADP is approximately 64–73% tetrameric, whereas in the other tested compounds, CtBP1 is 10–23% tetrameric for the data shown in Figure 1 (Table S1).

The elution volumes and MALS molecular mass values for CtBP2 show a similar pattern to those for CtBP1, with the notable exception of the response to AMP. In the presence of 10 μM NADH, 10 μM NAD^+^, and 1 mM ADP, the MALS measured molecular masses for CtBP2 are 169–176 kDa. CtBP2 in the presence of 1 mM AMP exhibits a MALS molecular mass of 132 kDa, only slightly higher than in the absence of any NAD moiety and much lower than with 1 mM ADP. For the the other tested compounds, the molecular masses range from 121 to 129 kDa, similar to 129 kDa for CtBP2 in the absence of any NAD moiety. Estimating the fractions of dimer and tetramer as described above, CtBP2 in NADH, NAD^+^, and ADP under these conditions is 63–73% tetramer. For the other tested compounds, CtBP2 is estimated to be 15–24% tetramer (Table S1).

### Concentration dependence of the ADP and AMP-linked assembly of CtBP1 and CtBP2

**Figure 2:**
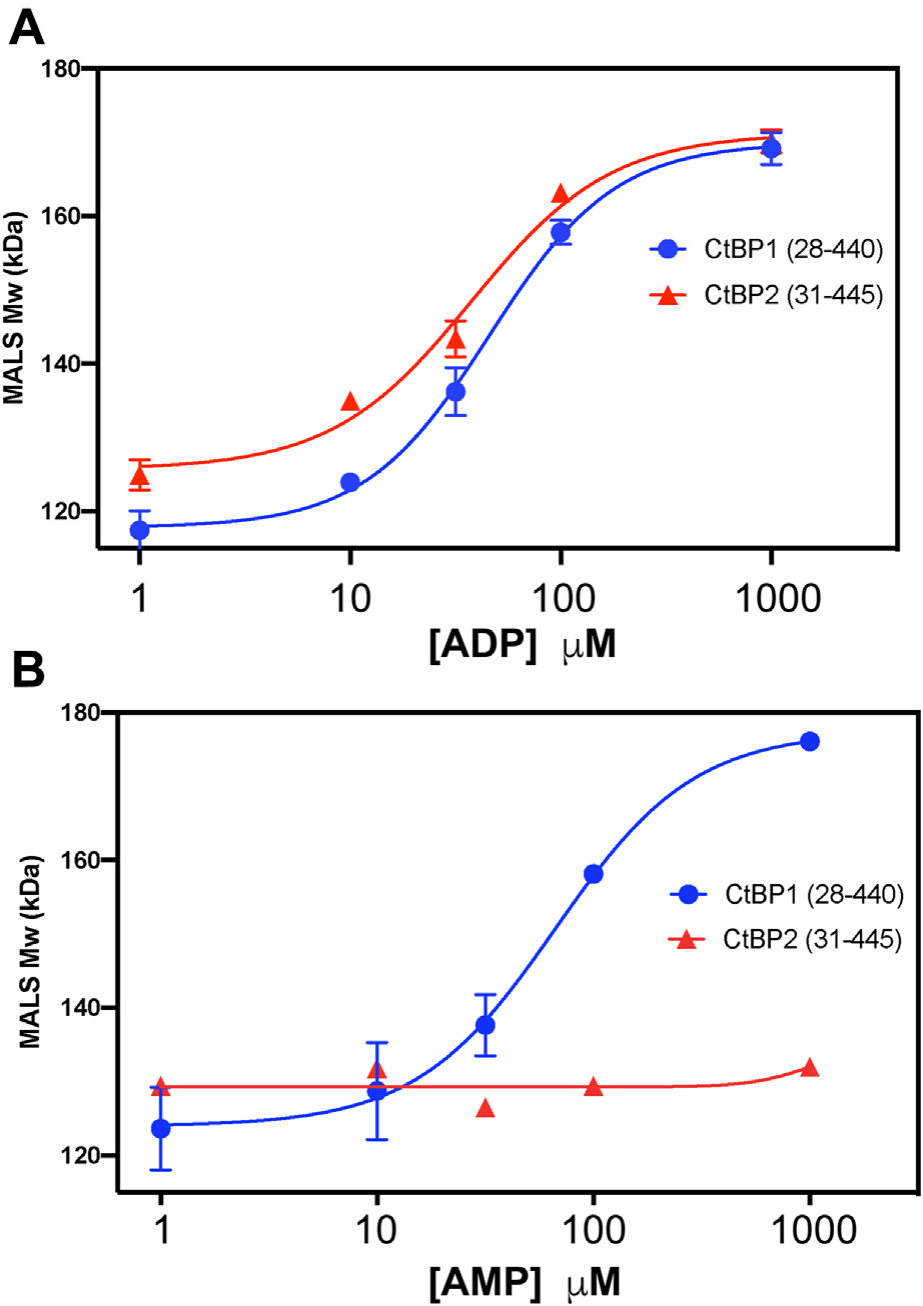
**Dependence of MALS-determined CtBP molecular masses as a function of ADP and AMP concentrations.** The data were fitted with Prism version 8 (see Experimental procedures). *A*, ADP dependent MALS-measured M_w_ values for CtBP1 (28–440) and CtBP2 (31–445). The data shown are for measurements of CtBP1 in triplicate and CtBP2 in duplicate. The *fitted curve* for CtBP1 indicates molecular masses of 118 kDa at low ADP and 170 kDa at high ADP, suggesting tetrameric fractions of 14% and 66%, respectively. The *fitted curve* for CtBP2 indicates molecular masses of 126 kDa at low ADP and 171 kDa at high ADP, suggesting tetrameric fractions of 19% and 66%, respectively. *B*, AMP-dependent MALS-measured M_w_ values for CtBP1 (28–440) and CtBP2 (31–445). The data shown are for measurements of CtBP1 in duplicate, but single measurements for CtBP2. The fitted curve for CtBP1 indicates molecular masses of 126 kDa at low AMP and 177 kDa at high AMP (with slightly higher protein concentrations than the ADP experiments) suggesting tetrameric fractions of 21% and 79%, respectively. (Individual data and additional replicates tabulated in Table S2.)

Previously we demonstrated that NADH and NAD^+^ promote tetrameric assembly of CtBP1 and CtBP2 with apparent EC_50_ values in the range of 50–150 nM (41). As discussed above, ADP promotes the tetrameric assembly of CtBP1 and CtBP2, whereas AMP promotes the tetrameric assembly of CtBP1. Figure 2 shows the measured MALS M_w_ values for CtBP1 and CtBP2 as a function of ADP and AMP concentration. (Individual results along with additional replicates are provided in Table S2.) Fitting the molecular mass dependence on ADP concentration (see Experimental procedures) indicates M_w_ values of 120 kDa and 126 kDa for CtBP1 and CtBP2, respectively, at low ADP concentrations rising to 170 kDa and 171 kDa at high ADP concentration for the measured conditions. The fitting yields an EC_50_ value for the effect of ADP promoting tetramer formation of about 52 μM for CtBP1 and 43 μM for CtBP2. Fitting the data for AMP yields an EC_50_ value of about 66 μM for CtBP1. Thus, although ADP and AMP (for CtBP1) promote tetramer formation as does NAD(H), the concentrations of AMP and ADP required for tetrameric assembly are approximately three orders of magnitude higher than required for NAD(H).

### Competition between NAD(H) and nontetramer inducing NAD moieties

**Figure 3:**
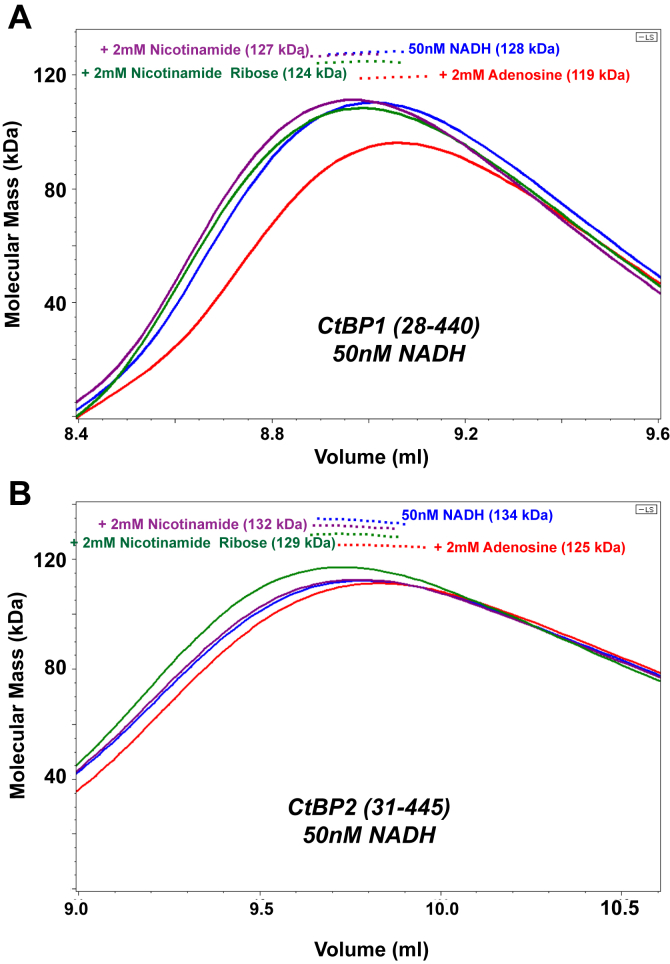
**Competition between 50 nM NADH and NAD moieties.***A*, CtBP1 and (*B*) CtBP2 SEC-MALS experiments with the column equilibrated with 50 nM NADH (*blue*), followed by 50 nM NADH plus 2 mM nicotinamide (*purple*), 2 mM nicotinamide ribose (*green*), or 2 mM adenosine (*red*). Adenosine showed the largest shift, lowering the observed molecular mass by 9 kDa under these conditions for both CtBP1 and CtBP2, with nicotinamide ribose and nicotinamide showing smaller, but consistent, shifts. For the experiments shown, the elution peak protein concentrations ranged from 1.6 to 1.7 μM, for CtBP1 and 0.59 to 0.63 μM, for CtBP2. (The column elution volumes for CtBP2 are generally larger than those of CtBP2, as evidenced in the difference between *A* and *B*. Individual data and replicates tabulated in Table S3.)

The inability of those NAD moieties that lack adenosine phosphate to promote CtBP tetramer formation could result from a lack of binding or binding that does not induce structural changes that stabilize tetramers. To test for specific binding, we investigated the ability of these compounds to interfere with NADH-linked tetramerization. Figure 3 shows SEC/MALS results for CtBP1 and CtBP2 in the presence of 50 nM NADH (slightly below the EC_50_ for tetramer formation (41)). Under these conditions, the MALS determined molecular masses of 128 kDa and 134 kDa for CtBP1 and CtBP2, suggesting approximately 22% and 25% tetramer, respectively. Addition of 2 mM adenosine reduces the molecular masses by 9 kDa, lowering the estimated tetramer fractions to 15% and 18%, respectively. Thus, adenosine is able to interfere with NADH-linked tetramer assembly, presumably by direct binding, which highlights the importance of the adenosine phosphate to promote tetramer formation. (The effect of adenosine showing lower MALS M_w_ values is also evident in Figure 1, suggesting a very small amount of residual bound NAD(H) may be present in those experiments, see below.) This series of experiments provides evidence that nicotinamide ribose and nicotinamide may also interfere with tetramer formation, but only to a very small extent. Although slight, the differences do appear to be reproducible (Table S3). These results suggest that adenosine, along with possibly nicotinamide and nicotinamide ribose, inhibits NADH-linked tetrameric assembly of CtBP by binding in the NADH pocket, albeit at much lower affinity. Thus, binding into the adenosine or nicotinamide ribose pockets is not sufficient to cause tetrameric assembly unless the adenosine phosphate is present.

### Structural investigation of AMP binding to CtBP1

**Figure 4:**
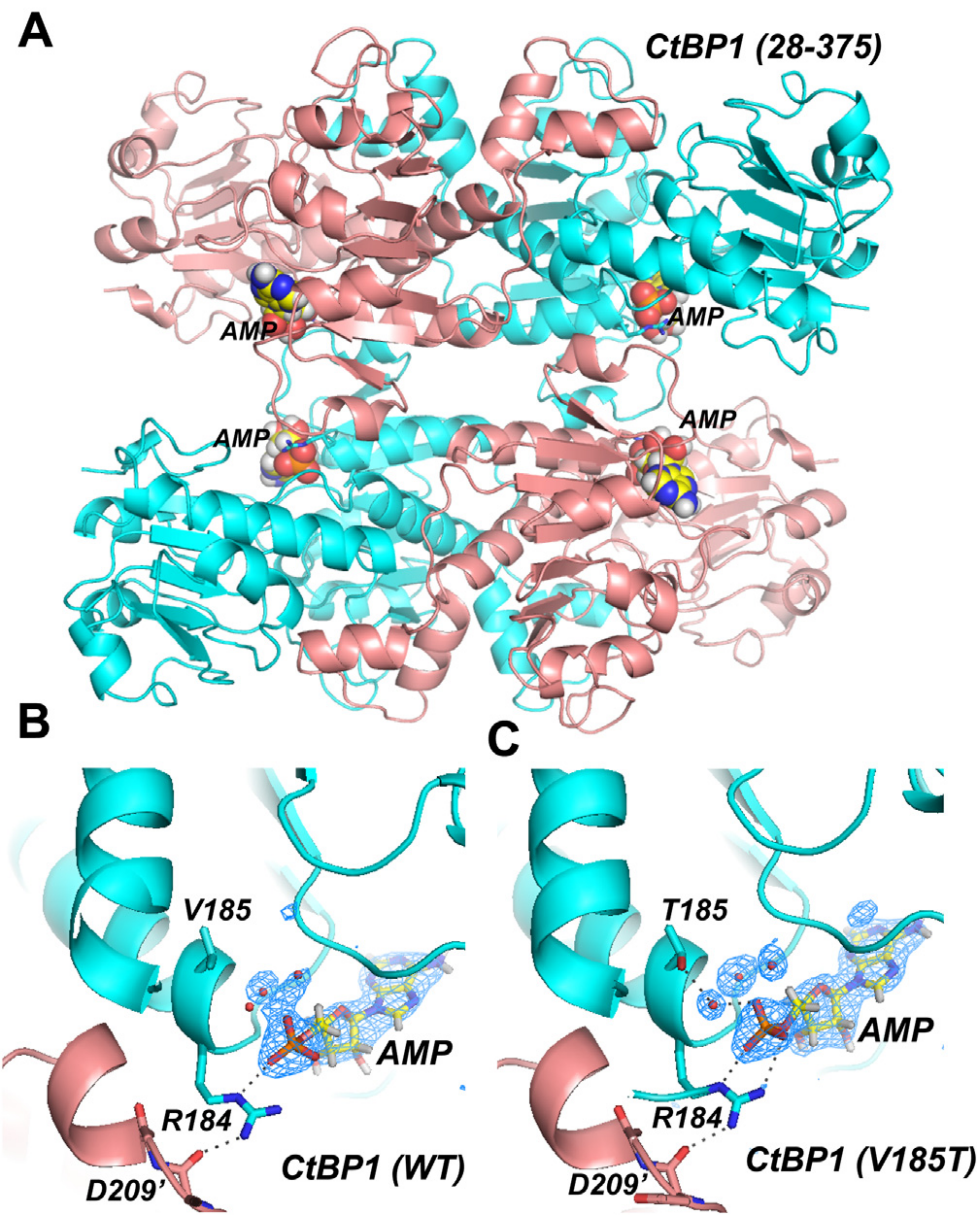
**AMP-bound crystal structures of CtBP1.***A*, trace of CtBP1 tetramer (WT, residues 28–375) with two subunits shown in cyan and two in salmon and the four bound AMP moieties shown as van der Waals spheres. *B*, omit F_o_-F_c_ electron density map, with AMP and neighboring water molecules removed, from CtBP1 (WT) AMP structure. Map is contoured at 3σ (*blue*) and clearly shows the AMP moiety, whose ionically stabilized hydrogen bond with arginine 184 positions it to form a hydrogen bond across the tetrameric interface with the main-chain carbonyl of Asp 209' of a neighboring subunit. (Apparent hydrogen bonds, with distances of 2.6 Å and 3.2 Å, are shown as *dashed lines*.) *C*, omit F_o_-F_c_ electron density map, with AMP and neighboring water molecules removed, from the CtBP1 (V185T) AMP structure. Map is contoured at 3σ (*blue*) and clearly shows the AMP moiety along with neighboring water molecules that are more well-ordered than in wild-type. It appears that a well-ordered water molecule stabilizes the AMP phosphate by forming bridging hydrogen bonds linking a phosphate oxygen with the mutant threonine hydroxyl (*dashed lines*, 2.6 Å and 2.7 Å), providing additional stabilization for Arg 184 and the tetrameric interface.

Our earlier crystal structures of CtBP1 and CtBP2 showed that the adenosine phosphate of NAD(H) interacts with a conserved Arg (184 in CtBP1, 190 in CtBP2), which then participates in a hydrogen bond with the main chain carbonyl oxygen of Asp 209′/215′ (CtBP1/CtBP2, primes designate a residue in a partner subunit) across the tetrameric interface (41, 44). (The similarity of this interaction in CtBP1 and CtBP2 is shown in Fig. S2.) To ascertain if AMP promotes tetramer assembly of CtBP1 in a similar fashion, we determined the crystal structure of CtBP1 (28–375) in complex with AMP at 2.45 Å resolution (Fig. 4*A*). (Crystallization of CtBP1 required the removal of the last 65 residues (41); CtBP1 (28–375) shows similar AMP-dependent tetramerization as does CtBP1 (28–440), Table S2.) As discussed in Experimental procedures, careful purification of CtBP1 (28–375) in the presence of 5 mM AMP was required to completely remove bound NAD(H) that is apparently acquired during expression in *E. coli*. Eventual success became clear from crystallographic analysis showing the electron density for AMP (Fig. 4*B*) but no observable density for the remainder of the NAD(H) cofactor.

The cocrystal structure of AMP bound to CtBP1 (28–375) confirms that AMP binds nearly identically to the AMP portion of NADH. In particular, the adenosine phosphate maintains charge-stabilized hydrogen bonding with Arg 184, which then forms a hydrogen bond across the tetrameric interface with the main-chain carbonyl oxygen of Asp 209 (Fig. 4*B*), which is nearly identical to that observed in CtBP1 and CtBP2 bound to NAD(H) (Fig. S2). Our finding that AMP triggers tetramer formation, but adenosine interferes with NADH-linked tetrameric assembly (Fig. 3), demonstrates that this phosphate is critical for tetramer formation, apparently by localizing Arg 184 for favorable hydrogen bonding across the interface.

### Distinct responses of CtBP1 and CtBP2 to AMP

**Figure 5:**
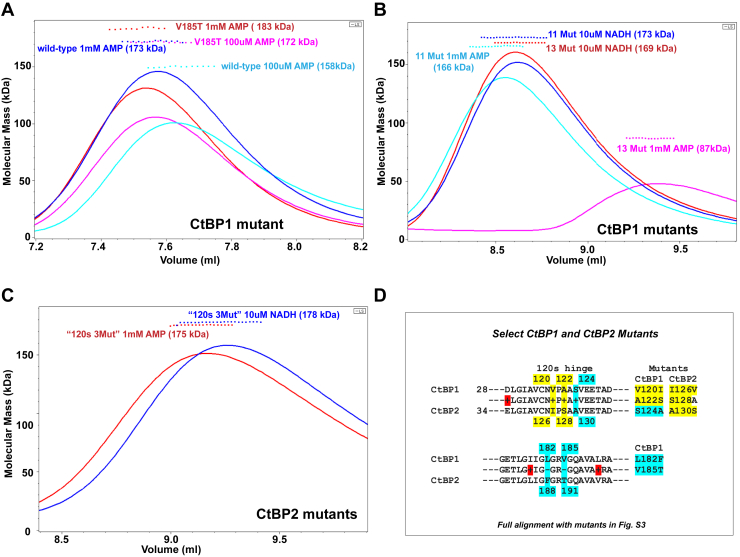
**SEC trace and MALS molecular masses showing the response of CtBP1 and CtBP2 mutants to AMP and NADH.** The *lines* show the light-scattering Rayleigh ratio (arbitrary units) for protein elution from the SEC column, and the small squares show the MALS molecular mass measurements across the elution peaks. *A*, traces comparing the mutant V185T with wild-type CtBP1 in 1 mM and 100 μM AMP. Note the substantially greater molecular masses for the V185T traces, indicating greater stabilization of the tetrameric form with bound AMP compared with wild-type. *B*, traces showing the CtBP1 11 mutant (L124A, L182F, V185T, V211I, A214S, L217V, G238N, T264A, E330A, K333T, K348R), which responds to 1 mM AMP and 10uM NADH similarly to wild-type. In contrast, the 13-mutant form of CtBP1 (adding V120I, A122S to the 11-mutant form) is much more similar to CtBP2 in its response to 1 mM AMP, exhibiting a primarily dimeric molecular mass (87 kDa) and to 10 μM NADH showing a primarily tetrameric molecular mass. *C*, traces of CtBP2 mutated to the CtBP1 residues in the “120s hinge” (I126V, S128A, A130S) respond to both 1 mM AMP and 10 μM NADH very similarly to CtBP1. (Individual data and replicates tabulated in Table S4. The difference in elution volumes for [*A*] and the others results from different columns.) *D*, partial alignment of CtBP1 and CtBP2 sequences showing key mutations constructed for the data shown in this figure, with NADH contacting residues highlighted in *cyan* and 120s loop residues highlighted in *yellow*. (Full sequence alignment and mutations provided in Fig. S3.)

To determine the basis for the distinct response of CtBP1 and CtBP2, we created a series of mutants at positions that have different residues in CtBP1 and CtBP2. Given the structural and sequence similarity between CtBP1 and CtBP2 (particularly in the dehydrogenase domain, with 88.7% amino acid sequence identity, Fig. S3), it was perhaps surprising to find such a striking difference in the response of these two proteins to AMP. (The minimal dehydrogenase domains of CtBP1 (28–353) and CtBP2 (31–364) showed similar AMP responses as the equivalent paralog with the full C terminus (Table S4) strongly suggesting that the basis for distinct responses resides within the minimal dehydrogenase domains.) We first explored those residues with sequence differences in contact with NADH. Five residues with different amino acid identity between CtBP1 and CtBP2 have atoms within 5 Å of NADH (Figs. S3 and S4). For each of the five, we mutated the CtBP1 residue to that in CtBP2 to determine the contribution of each to the distinct AMP responses. One particularly interesting mutant, V185T, enhanced tetramerization in the presence of AMP (Fig. 5*A*). The basis for the enhanced assembly of CtBP1 V185T became clear from the crystal structure, which revealed a well-ordered water molecule bridging the Thr hydroxyl and a phosphate on AMP in CtBP-V185T (Fig. 4*C*). This ordered water molecule stabilizes a cluster of water molecules in the vicinity of bound AMP, which apparently stabilizes bound AMP over that in wild-type CtBP1. The other single site mutants were similar to wild-type in their response to AMP, except for L182F, which is discussed in Figure S5. Combinations of these mutants did not convert CtBP1 into a form that displayed the observed CtBP2 response to AMP (Table S4). Thus, other residues must be responsible for the distinct AMP response of the two paralogs.

As a result, we widened the search of different residues between CtBP1 and CtBP2 to those in close structural proximity to the mutated coenzyme contacting residues and/or the tetrameric interface. Addition of six more residues created an 11-mutant form, termed “11Mut” (Fig. S3). 11Mut, along with various combinations of a smaller number of mutations, also did not convert CtBP1 into the AMP response observed in CtBP2 (Fig 5*C* and Table S4). However, adding two additional changes, V120I and A122S to 11Mut (which included an S124A mutation in the same peptide segment, Fig. 5*D*) reduced the molecular mass to slightly below that of a dimer, apparently eliminating tetramer formation in the presence of AMP while maintaining primarily tetrameric form in the presence of NADH (Fig. 5*B*). Thus, CtBP1 with these 13 mutations approximates the AMP and NADH behavior of CtBP2.

**Figure 6:**
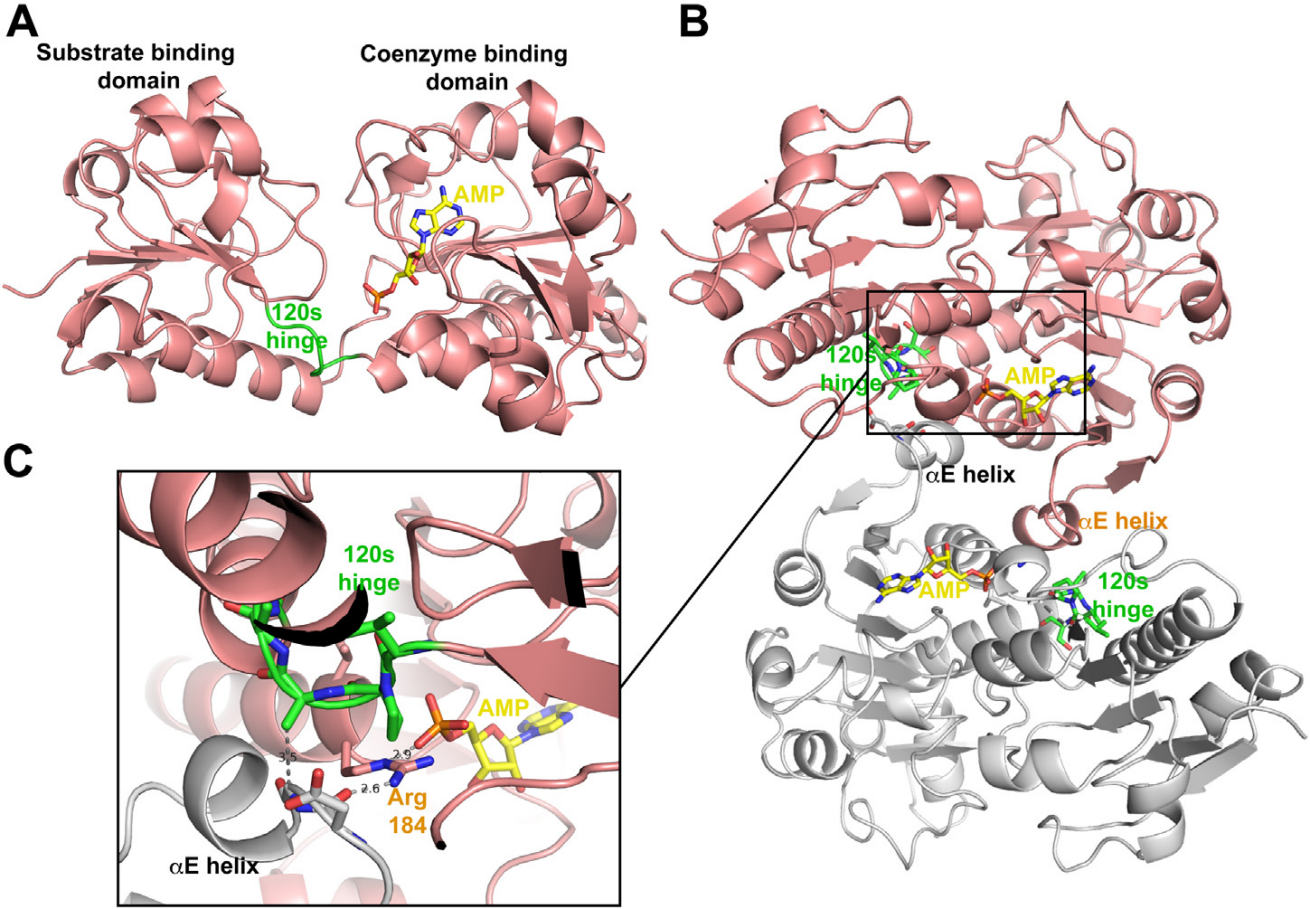
**Structure of CtBP1-AMP highlighting the 120s hinge.***A*, one subunit showing the two domains spanned by the 120s hinge (*green*). *B*, two subunits contacting at the tetrameric interface. Note the short αE helix in each subunit projecting toward the partner subunits making contacts with the 120s hinge residues. *C*, tetrameric interactions in the region of the 120s hinge. Note the close proximity of the 120s hinge to the short αE helix of the partner subunit and to the critical Arg 184, which bridges between the AMP phosphate and partner subunit.

This search identified a key peptide segment, including V120, A122, and the neighboring coenzyme contacting Ser124 as important in the response of AMP binding. This segment, which we refer to as the “120s hinge,” is one of two segments that connect the substrate and coenzyme-binding domains (Fig. 6*A*). In the 120s hinge, Ser 124 contacts NADH and Ala 122 makes contacts in the tetrameric interface (Fig. 6), and thus this hinge region is in a critical position to both sense binding in the coenzyme pocket and contribute to tetrameric assembly. In the context of wild-type CtBP1, triple mutation of just the 120s hinge is dimeric (92 kDa) in the presence of 1 mM AMP, but only partially tetrameric (142 kDa) in 10 μM NADH (Fig. S5). Thus, mutation of the 120s hinge residues is necessary for weakening the AMP response, but not sufficient to approximately match the complete CtBP2 response.

The centrality of the 120s hinge was tested by creating the converse mutants in CtBP2. Mutation of I126V, S128A, and A130S (Fig. 5*D*) resulted in protein that maintained a primarily tetrameric arrangement in the presence of both 1 mM AMP and 10 μM NADH (Fig. 5*C*). Thus, mutation of just these three residues is sufficient to convert CtBP2's response to AMP and NADH to be similar to that of CtBP1. These results demonstrate that the 120s hinge segment is central for the distinct response of the two CtBP paralogs.

## Discussion

NAD(H) is involved in both cotranscriptional activity and oligomerization of CtBP (5, 29, 33, 36, 37). Although oligomerization has most often been considered as dimerization, we (41) and others (34) have demonstrated that the primary effect of NAD(H) is to promote assembly of CtBP dimers into tetramers. Moreover, we have recently established the transcriptional relevance of the tetrameric forms by showing that tetramer-destabilizing mutants of CtBP2 are defective for oncogenic activity (42). Our results here have identified the phosphate groups as central for NAD(H)-linked assembly of tetrameric CtBP and also identified an important difference between CtBP1 and CtBP2 that emanates from a short peptide segment that connects the two subdomains. These findings are key for defining how CtBP exerts its cotranscriptional effects and for efforts to design inhibitors that could become lead compounds for antineoplastic agents.

The results presented here provide new insight into the trigger for CtBP oligomerization. Madison *et al*. (34) proposed that the binding of NAD(H) triggered the formation of a dimer as a result of a hydrogen bond between Trp 318 and the nicotinamide. Dimeric assembly is a prerequisite for assembly of two dimers into a tetramer (34, 41). The conformation of Trp 318 is essentially identical in CtBP1 bound to NAD^+^ and AMP lacking the nicotinamide moiety (Fig. S7), arguing against the hypothesis that a direct interaction between the nicotinamide and Trp 318 is key for NAD(H) triggered assembly of CtBP. Moreover, our finding that AMP or ADP can trigger assembly strongly suggests that interactions with the nicotinamide ring are dispensable for dimer and tetramer formation, as nicotinamide, nicotinamide ribose, and nicotinamide nucleotide all fail to trigger CtBP oligomerization. Our light scattering experiments, both presented here and earlier (41), strongly suggest that CtBP1 and CtBP2 assemble into dimers even in the absence of NAD(H), which is not surprising given the very extensive dimeric interface (∼2500 A^2^). Rather, our evidence indicates that it is the interaction of the adenosine phosphate with Arg 184/190 that is the key moiety triggering tetrameric assembly upon NAD(H) binding.

Substantial evidence (4, 15, 16, 17, 18, 19, 20, 21, 22, 23, 24, 25, 26) suggests that CtBP may be a useful target for antineoplastic agents. Application of structure-based drug design approaches requires a detailed understanding of cotranscriptional activation, which for CtBP includes defining the trigger for tetramer formation. In particular, it is important that agents designed to inhibit NADH binding do not inadvertently promote oligomerization. Our results demonstrate that binding of ligands in the coenzyme pocket does not promote tetramer formation unless the adenosine phosphate is present. Thus, other portions of the coenzyme pocket can be exploited in structure-based drug design to interfere with NAD(H) binding and disrupt CtBP cotranscriptional activity in cancer.

Our earlier structural analysis of CtBP1 and CtBP2 (44) revealed a hydrophilic cavity linking the substrate MTOB (4-methylthio 2-oxobutyric acid) with the pocket binding nicotinamide ribose and phosphate (Fig. S8). Targeting this cavity for inhibitor design should confer specificity, as this cavity is unique to CtBP1 and CtBP2. Our results here showing that nicotinamide and nicotinamide ribose do not trigger tetramer formation suggests a specific strategy for structure-based drug design. Compounds could be designed that both bind with high affinity in the substrate pocket (as we have previously demonstrated (45)) and extend through the hydrophilic cavity into the nicotinamide pocket to inhibit NADH binding.

Functional differences between CtBP1 and CtBP2 are evident; for instance, CtBP2 knockouts are embryonically lethal in mice, whereas the effect of knocking out CtBP1 is more subtle (6). Such differences may extend to tumor cells as well, in which case eventually developing compounds with specificity for CtBP1 or CtBP2, but not both, may be advantageous. As a result, it is important to delineate differences even in proteins as structurally similar as CtBP1 and CtBP2.

The similar NAD(H) concentration dependence for CtBP1 and CtBP2 assembly into tetramers (41) suggests similar coenzyme affinity. However, the distinct responses to AMP identified here highlight an important difference. Our results demonstrate that a short segment connecting the two CtBP domains, which we term the 120s hinge, is primarily responsible for distinct paralog responses to AMP binding. The proximity of the 120s hinge to both the substrate binding pocket and the tetrameric interface suggests that it could be possible to target this region to obtain paralog-specific inhibitors. Our results also highlighted another important sequence variation, Thr 191 in CtBP2, which is occupied by the isosteric, but nonpolar, Val 185 in the homologous position in CtBP1. It is likely that the hydrogen bond between Thr 191 and the nicotinamide phosphate of NAD(H) contributes to the stabilization of coenzyme binding in CtBP2 that is absent in CtBP1. This interaction is most likely formed in ADP, which shows similar concentration dependence for both CtBP2 and CtBP1, but is absent in AMP. Thus, the hydrogen bond between Thr 191 and the nicotinamide phosphate is likely to be important for coenzyme binding in CtBP2. Therefore, one approach to CtBP2-specific inhibitors could include a moiety that can directly interact with Thr 191 to favor inhibition of CtBP2 over CtBP1.

In conclusion, our studies demonstrate that the linkage between NAD(H) binding and CtBP tetramer formation requires the adenosine phosphate of the coenzyme. These results highlight the nicotinamide ribose pocket as a favorable receptor for inhibitors whose binding should disrupt tetramer formation and cotranscriptional activity. An understanding of the stereochemical details for the assembly of CtBP dimers into tetramers can, thus, contribute to the development of highly specific inhibitors of CtBP in cancer.

## Experimental procedures

### Expression, purification, and mutagenesis of CtBP1 and CtBP2

The expression and purification procedures followed those of earlier studies (41, 44, 45). CtBP1 and CtBP2 mutants were created with the QuikChange protocol (Strategene) using the modified approach of Liu and Naismith (46). The final step in purification was an FPLC size-exclusion column (Highload 16/60 Superdex 200 preparation grade) carried out in the presence of 10 μM NADH. During our analysis of various mutants, a prescreen for their response to AMP was gained from a second run on this column in the presence of 1 mM AMP, which provided an early classification of whether mutants behaved more like CtBP1 (with similar elution volumes in both NADH and AMP) or CtBP2 (with later elution in the presence of AMP). These results were consistent with the more precise MALS results reported.

### SEC-MALS studies for oligomerization

The CtBP constructs explored by SEC-MALS include the full C-terminal region—CtBP1 (residues 28–440) and CtBP2 (residues 31–445), each with a six-histidine tag at the amino terminus. Protein samples were prepared for SEC-MALS by diluting CtBP stocks to approximately 1.0 mg/ml in SEC-MALS running buffer (50 mM HEPES, pH 7.4, 300 mM NaCl, 1 mM EDTA, 2 mM DTT). The protein samples were filtered with a Costar 0.22 μm Spin-X column at room temperature. After full equilibration of the system with the SEC-MALS running buffer supplemented by various concentrations of NAD(H) moieties, 100 μl protein samples was injected into the instrument. The SEC-MALS system consisted of a Dawn Helios-II MALS detector (Wyatt), an Optilab T-rEX differential refractive index detector (Wyatt), and the 1260 Infinity HPLC system (Agilent) with a TSKgel G3000SWxl column (Tosoh Bioscience). The experiments performed were obtained from four different columns, as CtBP use tended to degrade the columns after 50–100 runs. Cleaning the columns with 5 M urea improved the column function, however, with a loss of some resolution in protein separation and variation in the concentration of protein elution. As a result of column variability, the data reported in each figure were derived using the same column and protein stock and were collected within 2 days of each other for direct comparison.

The data for dependence of the molecular mass as a function of AMP and ADP concentration (Fig. 2) were fit with Prism 8 (GraphPad Software, Inc) to the equation $Y=L+\left(U-L\right)/\left(1+10^{\text{log}\left(EC50-x\right)n}\right)$, where L and U are the lower and upper M_w_ plateaus, respectively, x is the concentration of AMP/ADP in μM, and n is the Hill coefficient.

The molecular mass obtained from light scattering from a heterogeneous mixture of protein molecules is the weight average molecular weight (M_w_): M_w_ = ΣN_i_M^2^_i_/ΣN_i_M_i_, where N_i_ is the molar or fractional concentration (43). Assume that a mixture of just CtBP dimers and tetramers allows simplification to M_w_ = [F_T_(2M_D_)^2^ + (1 − F_T_)_i_M_D_^2^]/[F_T_(2M_D_) + (1 − F_T_)M_D_], where M_D_ is the dimeric molecular weight and F_T_ is the fraction of CtBP in the tetrameric form. Rearranging this equation, we obtained estimates of the fraction tetramer from F_T_ = (M_w_ − M_D_)/(3M_D_ − M_w_).

### Crystallization and X-Ray diffraction

Our initial crystallographic experiments to obtain an AMP-bound CtBP1 (28–375) structure demonstrated clear electron density indicating bound NAD(H), albeit at less than full occupancy. Evidently, NAD(H) acquired during bacterial expression remained bound to CtBP1 during purification. Eventually we were able to completely remove NAD(H) (definitively shown by final electron density maps, Fig. 5*B*) by carrying out purification in the presence of 5 mM AMP. The final, crucial, step was a size-exclusion run on an FPLC (ÄTKAprime plus by GE Healthcare) using a size exclusion column (Highload 16/60 Superdex 200 prep grade) equilibrated with buffer comprising 50 mM Tris:HCl, pH 7.7, 300 mM NaCl, 5 mM EDTA, 2 mM DTT, and 5 mM AMP. Similar procedures were carried out for the purification of AMP-bound CtBP1 V185T (28–375) and CtBP1 L182F/L217V (28–375).

Following purification, the protein was maintained in 5 mM AMP throughout, concentrated to 8–12 mg/ml and supplemented with 2 mM tris(2-carboxyethyl) phosphine (TCEP). The sample was then filtered with a Costar 0.22 μm Spin-X column at room temperature. Hanging vapor diffusion drops were set up in a 1:1 ratio of protein to mother liquor with a total volume of 4–8 μl and incubated at 20 °C. Crystals formed within 24 h but were allowed to grow for several days. Suitable crystals with hexagonal bipyramidal morphology grew from reservoir conditions of 100 mM HEPES buffer pH 7.5 containing 80–200 mM CaCl_2_ and 2–6% PEG400. A cryogenic solution was prepared containing 100 mM HEPES buffer, pH 7.5, 200 mM CaCl_2_, 12% PEG400, and 12% ethylene glycol. Crystals were equilibrated with this solution by vapor diffusion for several hours before harvesting by adding the cryo solution directly to the crystals, mounting, and flash-freezing at 100K. Diffraction data from wild-type and V185T mutant crystals were collected on a home source MicroMax-007-HF/Saturn 944 CCD X-ray diffraction system (Rigaku) and then processed with HKL-3000R (47). Diffraction data from the L182F/V185T double mutant were kindly collected by Dr Mark Del Campo on a HyPix-6000HE HPC detector at Rigaku Oxford Diffraction. The structures were determined by molecular replacement, using the CtBP1 (28–378) crystal structure, PDB ID code 6CDF (41) as the search molecule, and refined in PHENIX (48). Model building between rounds of refinement was performed using COOT (49). Refinement was carried out to a resolution of 2.45 Å for wild-type, 2.35 Å for the V185T mutant and 2.3 Å for the L182F/V185T mutant (Table 1).

**Table 1:** Crystallographic data collection and refinement statistics

	CtBP1 (28–375) WT-AMP	CtBP1 (28–375) V185T-AMP	CtBP1 (28–375) L182F/V185T-AMP
PDB Code	6V89	6V8A	7KWM
Data Collection			
Space Group	P6_4_22	P6_4_22	P6_4_22
a, b, c (Å)	88.94, 88.94,163.7	89.17, 89.17, 164.2	89.26, 89.26, 163.7
α, β, γ (°)	90, 90, 120	90, 90, 120	90, 90, 120
Bragg Spacings(Å)^a^	35.–2.45 (2.54–2.45)	50.–2.35 (2.43–2.35)	50.–2.3 (2.38–2.3)
R_merge_	0.091 (0.539)	0.107 (0.938)	0.108 (1.07)
Mean I/σ_I_	14.4 (1.8)	16.3 (1.2)	17.65 (1.78)
Completeness (%)	92.4 (71.9)	99.4 (98.0)	99.4 (97.5)
Redundancy	7.7 (2.8)	9.4 (3.9)	7.4 (4.2)
Total Reflections	107453 (2889)	157667 (6173)	131678 (7323)
Unique Reflections	13,944 (1028)	16,722 (1581)	17,800 (1723)
CC ½	0.998 (0.766)	0.998 (0.59)	0.994 (0.22)
Refinement			
R_work_/R_free_	0.231/0.274	0.203/0.258	0.211/0.260
# Nonhydrogen Atoms			
Protein	2535	2506	2536
AMP	23	23	23
Solvent	152	220	110
Mean B-Factors (Å^2^)			
Protein	49.6	40.4	48.1
AMP	50.6	32.8	
Solvent	49.2	46.6	46.1
r.m.s. Deviations			
Bond Lengths (Å)	0.002	0.002	0.004
Bond Angles (°)	0.48	0.46	0.69
Ramachandran (%)			
Favored	96.4	96.7	97.0
Allowed	3.6	3.3	3.0
Outliers	0	0	0
No. TLS Groups	1	1	1

a Highest resolution shell shown in parenthesis.

## Data availability

The structure factors and coordinates for the crystal structures described here have been deposited in the Protein Data Bank (https://www.rcsb.org) with accession codes 6V89 (CtBP1 [28–375] AMP), 6V8A (CtBP1 [28–375] V185T AMP), and 7KWM (CtBP1 [28–375] L182F/V185T AMP). All other data are contained within the document.

## Conflict of interest

The authors declare that they have no conflicts of interest with the contents of this article.
