# Supplementary material for: NAD(H) phosphates mediate tetramer assembly of human C-terminal binding protein (CtBP)
Source: J Biol Chem. 2021 Jan 30;296:100351. doi: 10.1016/j.jbc.2021.100351 (PMC7949142; doi:10.1016/j.jbc.2021.100351)
Supplement: Figures S1 to S8 and Tables S1 to S4 [file mmc1.pdf]

Supporting Information

NAD(H) phosphates mediate tetramer assembly of human C-terminal binding protein (CtBP)

Jeffry C. Nichols[1,2], Celia A. Schiffer[1] and William E. Royer, Jr.[1*]

From the [1]Department of Biochemistry & Molecular Pharmacology, University of Massachusetts Medical School, Worcester, MA 01605; [2] Chemistry Department, Worcester State University, Worcester MA 01602

Supporting information includes eight figures (S1- S8) and four tables (S1 – S4) which provide supporting structural information and details from multiangle light scattering (MALS) results presented in the main text and figures.

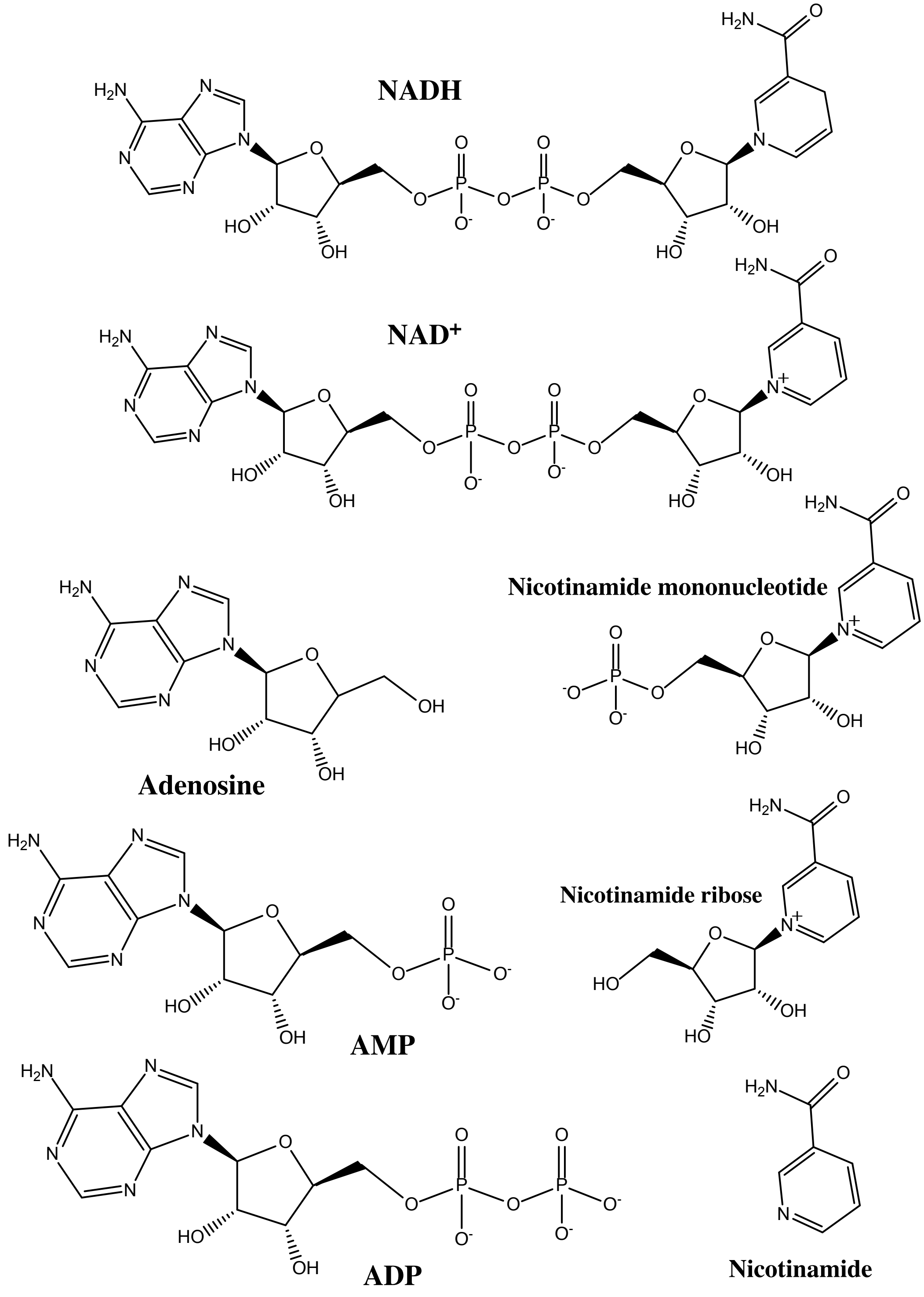

Figure S1. Chemical structures of NAD(H) and moieties investigated in this study.

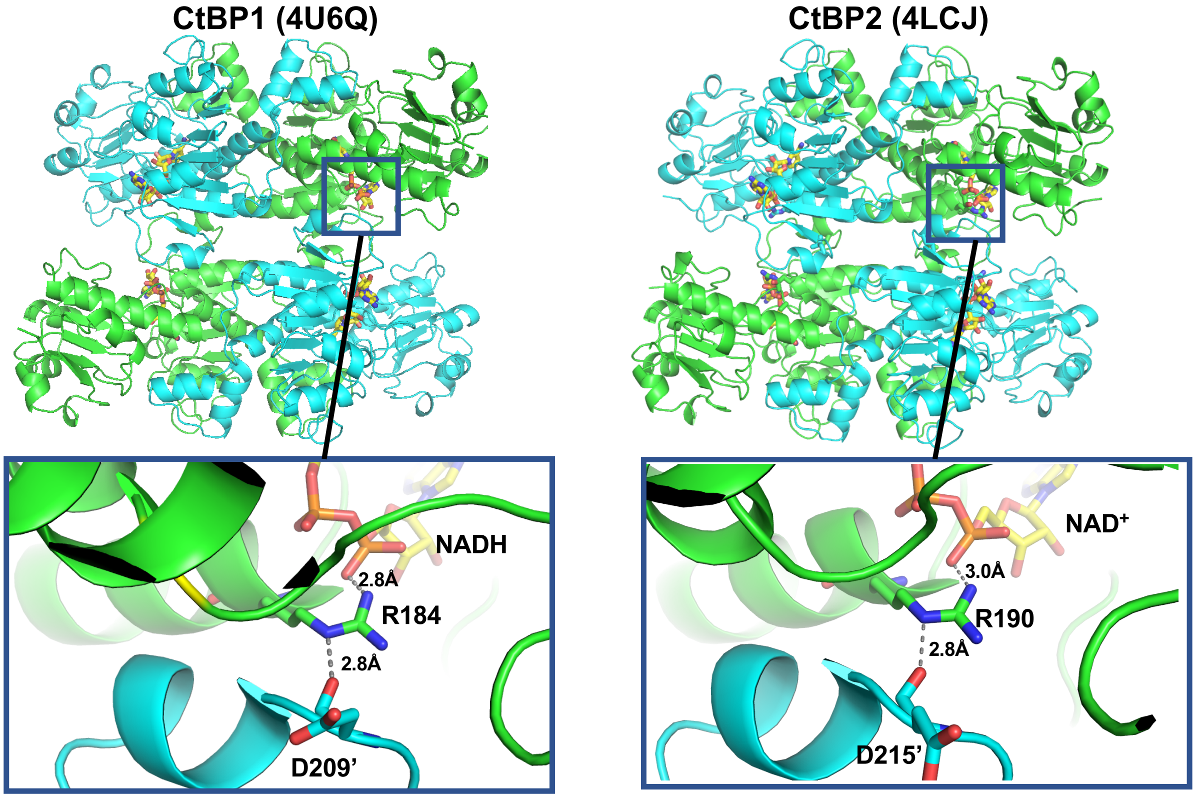

Figure S2. Comparison of the Arg 184/190 interaction across the tetrameric interface in CtBP1 and CtBP2. Top images show ribbon diagram of the tetramer structure with two subunits in green and two subunits in cyan for CtBP1 (left) and CtBP2 (right) along with a stick representation of NAD(H) with yellow carbon atoms. Bottom images show an expanded view of the interaction Arg 184 (CtBP1) and Arg 190 (CtBP2) across the tetrameric interface, including hydrogen bond distances. Note the similar interactions of this arginine residue bridging the adenosine phosphate with the carbonyl oxygen of Asp 209'/215' to contribute to the tetrameric interface.

```
        28                  48                        74
CtBP1    PLVALLDGRDCTVEMPILKDVATVAFCDAQSTQEIHEKVLNEAVGALMYHTITLTREDLE
         PLVALLDGRDCTVEMPILKD+ATVAFCDAQSTQEIHEKVLNEAVGA+MYHTITLTREDLE
CtBP2    PLVALLDGRDCTVEMPILKDLATVAFCDAQSTQEIHEKVLNEAVGAMMYHTITLTREDLE
        34                  54                        80
                                      120s hinge
             94     102 105 109 112  120 122 124   133       143
CtBP1   KFKALRIIVRIGSGFDNIDIKSAGDLGIAVCNVPAASVEETADSTLCHILNLYRRATWLH     V120I
        KFKALR+IVRIGSG+DN+DIK+AG+LGIAVCN+P+A+VEETADST+CHILNLYRR-TWL+     A122S
CtBP2   KFKALRVIVRIGSGYDNVDIKAAGELGIAVCNIPSAAVEETADSTICHILNLYRRNTWLY     S124A
            100     108 111 115 118  126 128 130   139       149

                                    179 182 185  191    199 201
CtBP1  QALREGTRVQSVEQIREVASGAARIRGETLGIIGLGRVGQAVALRAKAFGFNVLFYDPYL      L182F
       QALREGTRVQSVEQIREVASGAARIRGETLG+IG-GR-GQAVA+RAKAFGF+V+FYDPYL      V185T
CtBP2  QALREGTRVQSVEQIREVASGAARIRGETLGLIGFGRTGQAVAVRAKAFGFSVIFYDPYL
                                    185 188 191  197    205 207

        211 214 217 221 228 229 234 238          252          264        V211I
CtBP1  SDGVERALGLQRVSTLQDLLFHSDCVTLHCGLNEHNHHLINDFTVKQMRQGAFLVNTARG      A214S
       -DG+ER+LG+QRV-TLQDLL+-SDCV+LHC-LNEHNHHLINDFT+KQMRQGAFLVN-ARG      L217V
CtBP2  QDGIERSLGVQRVYTLQDLLYQSDCVSLHCNLNEHNHHLINDFTIKQMRQGAFLVNAARG      G238N
        217 220 223 227 234 235 240 242          258          270        T264A

                                      300             316      325
CtBP1  GLVDEKALAQALKEGRIRGAALDVHESEPFSFSQGPLKDAPNLICTPHAAWYSEQASIEM
       GLVDEKALAQALKEGRIRGAALDVHESEPFSF+QGPLKDAPNLICTPH-AWYSEQAS+EM
CtBP2  GLVDEKALAQALKEGRIRGAALDVHESEPFSFAQGPLKDAPNLICTPHTAWYSEQASLEM
                                      306             322      331

       330 333        345 348  353
CtBP1  REEAAREIRRAITGRIPDSLKNCVNK       E330A
       RE-AA-EIRRAITGRIP+SL+NCVNK       R333T
CtBP2  REAAATEIRRAITGRIPESLRNCVNK       K348R
       336 339        351 354  359
```

Figure S3. Alignment of minimal dehydrogenase domains of CtBP1 and CtBP2. Numbers above and below the sequences provide the residue numbers for those different between CtBP1 and CtBP2, respectively. Highlighted are the first five mutants created of residues within 5Å of NADH (cyan), the next six residues mutated (green), the additional two 120s hinge mutants (yellow) and the CtBP1/2 sequence differences not mutated (red). The "11 Mut" form, discussed in the manuscript and Fig.5, includes all mutations highlighted in green and cyan, whereas the "13 Mut" form includes those 11 mutations plus the two highlighted in yellow. (Residue color coding used here are the same as in Fig. S4 showing residue locations in the CtBP1 three-dimensional structure.)

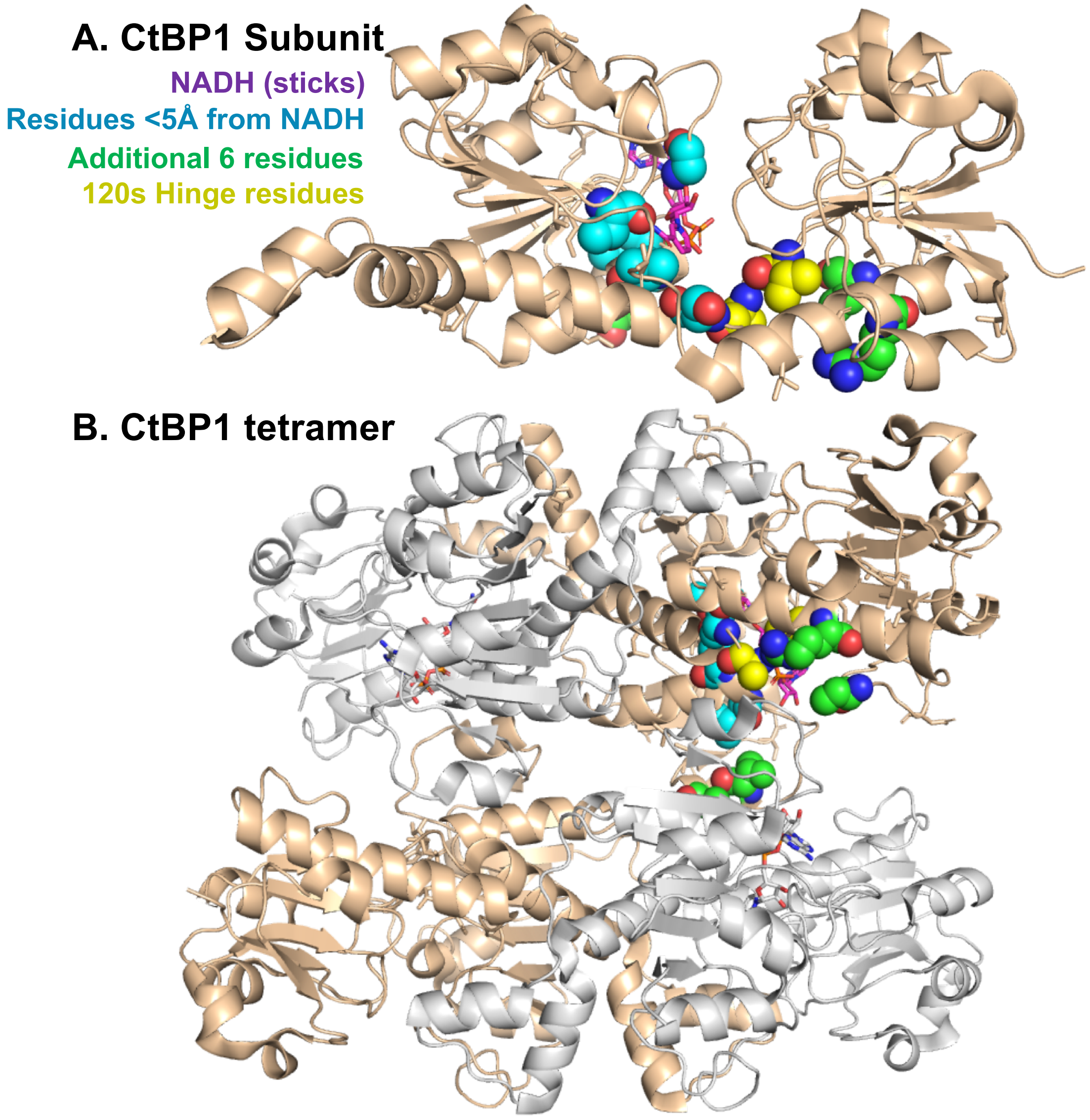

Figure S4. Location of mutated residues in three-dimensional CtBP1 structure. *A,* CtBP1 subunit (pdb ID 4U6Q) highlighting NADH (purple sticks) and the CtBP1 to CtBP2 mutants constructed. (For all, oxygen atoms are shown as red, nitrogens as blue and phosphorous as orange with carbon atoms in colors based on mutant group.) Three different groups of mutants are shown: residues within 5Å of NADH (S124A, L182F, V185T, G238N and T264A, shown with cyan spheres), 120s hinge mutants (V120I and A122S shown with yellow spheres, along with S124A shown in cyan) and six additional mutants (V211I, A214S, L217V, E330A, R333T, K348R, shown with green spheres). *B,* CtBP1 tetramer, with the subunit represented in (A) with mutants and trace shown as wheat background along with the three other tetrameric subunits, two of which shown as gray traces and one with a wheat trace. (Mutant color coding used here is the same as in Fig. S3 showing sequence differences.)

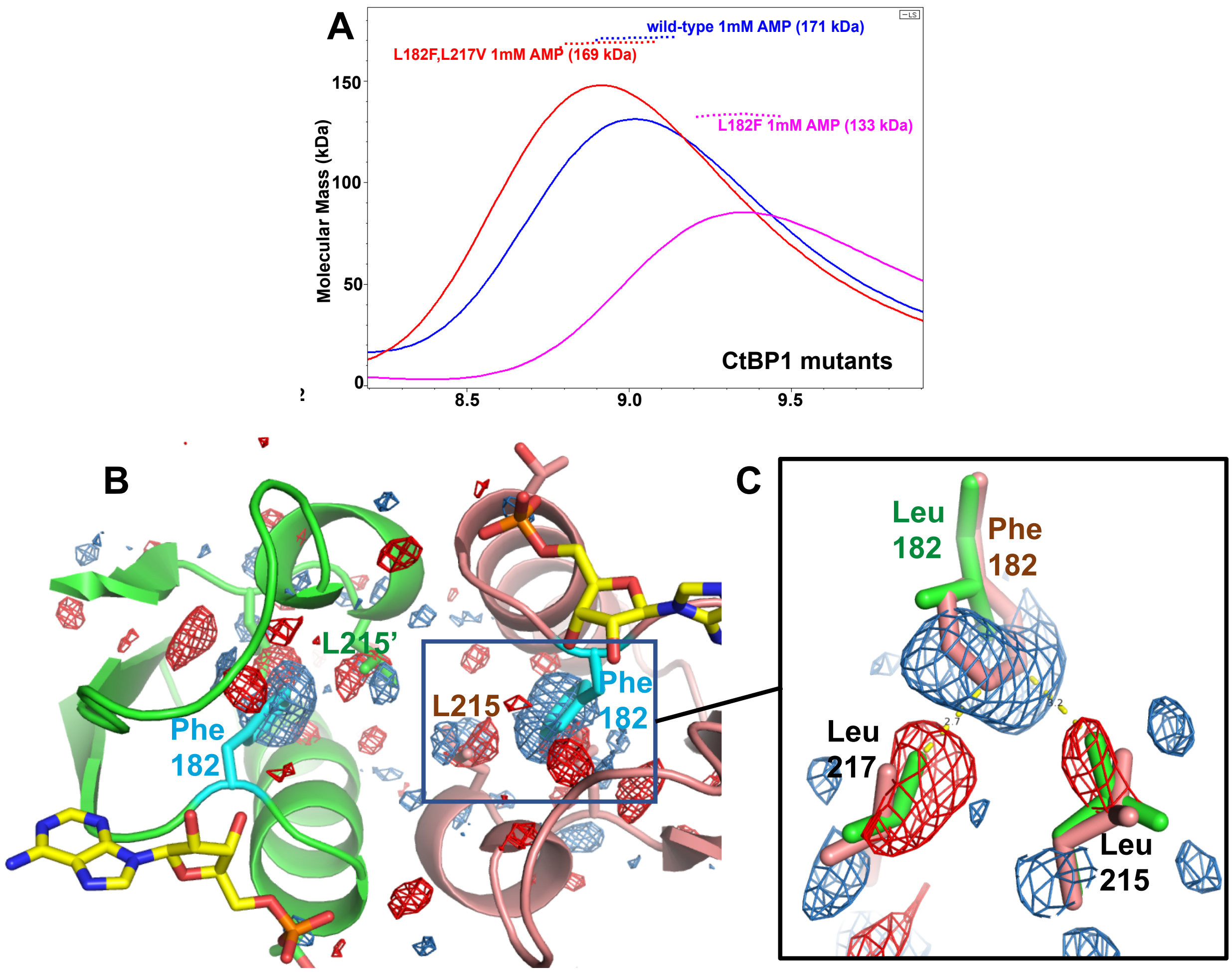

Figure S5. Structural basis for the AMP binding effect of the NADH adjacent L182F mutation. *A,* SEC-MALS traces for wild-type CtBP1, mutant L182F and double mutant L182F/L217V. Mutation of L182F reduces the molecular mass of CtBP1 in the presence of AMP. *B,* and *C*, F_o-F_o maps between CtBP1 (L182F/V185T) and CtBP1 (V185T). Density maps are contoured at +4σ blue, -4σ red. Blue contours clearly show the additional electron density for the phenyl ring in the Phe 182 mutant. Negative density on the side-chains of Leu 215 and Leu 217, along with the slight rotation of the two side chains in the mutant, shows the steric effect of adding a bulky Phe at position 182. The effect of this change on Leu 215 at the tetrameric interface (B) explains the weakened tetramer formation in the L182F mutant (A). Mutating Leu 217 to Val (the residue at this position in CtBP2) relieves this steric stress such that the double L182F/L217V is primarily tetrameric in the presence of 1mM AMP (A).

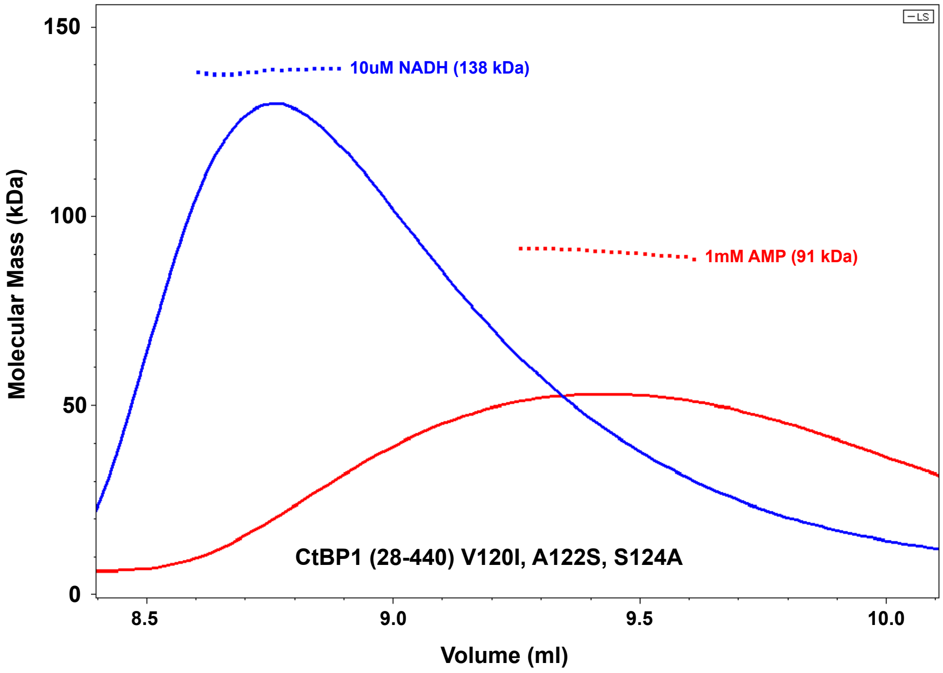

Figure S6. SEC trace and MALS molecular masses showing the response of CtBP1 (28-440) with just the 120s mutations (V120I, A122S, S124A) to NADH and AMP. The lines show the light-scattering Rayleigh ratio (arbitrary units) for protein elution from the SEC column and the small squares show the MALS molecular mass measurements across the elution peaks. This mutant shows a molecular mass (138 kDa) suggesting a mixture of about one-third tetramer and two-thirds dimer in the presence of 10uM NADH and a fully dimeric molecular mass (91 kDa) in the presence of 1mM AMP. Thus, these three mutations in the 120s hinge substantially weaken the tetramer in the presence of NADH and essentially eliminates tetramer formation in the presence of AMP. These results, combined with those in the main text, indicate that mutation of these three residues in the 120s loop is necessary, but not sufficient, to convert the behavior of CtBP1 to that of CtBP2 in AMP and NADH.

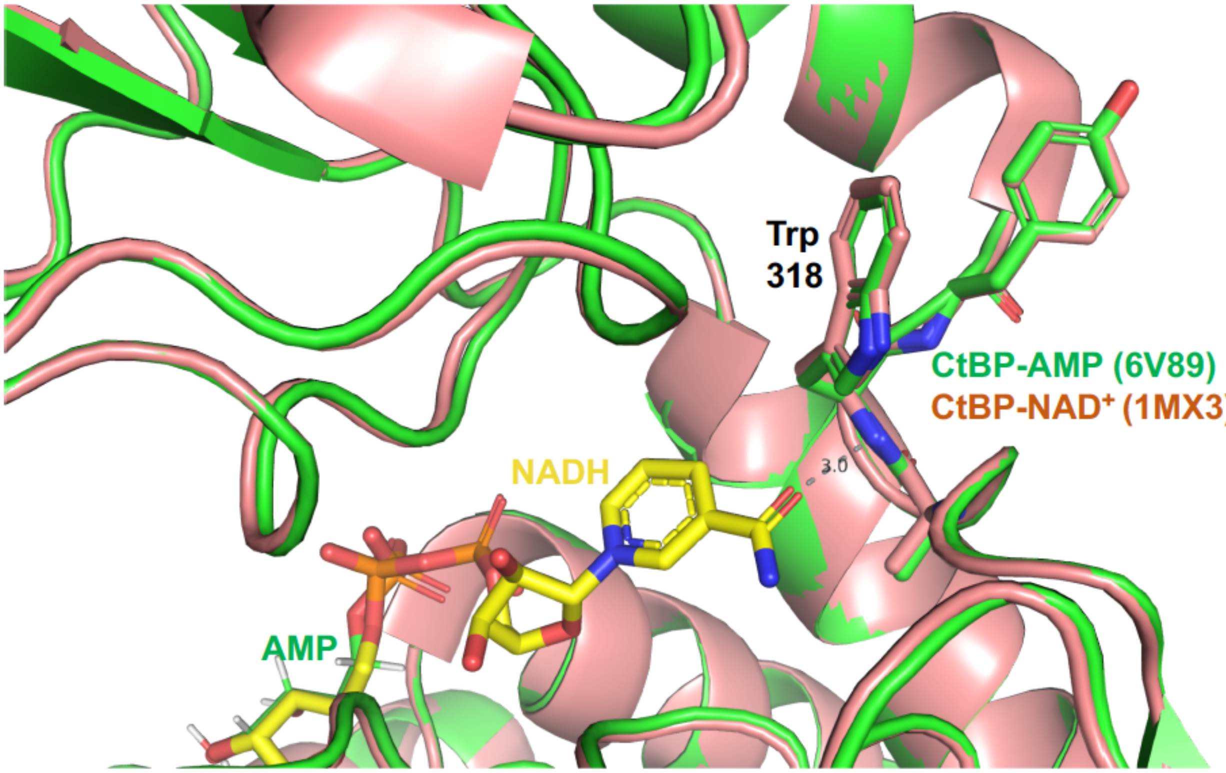

Figure S7. Comparison of the conformation of tryptophan 318 in AMP and NAD$^+$ co-crystal structures. Shown is the region around Trp 318 along with bound nucleotides in two CtBP structures, one with bound NAD$^+$ (Kumar *et al.*, 2002 Mol Cell. **10**, 857-869) and one with bound AMP. The presence or absence of the nicotinamide ring and the hydrogen bond to the amide nitrogen of Trp 318 has no significant effect on conformation of this residue, arguing against the hypothesis that this hydrogen bond plays a key role in triggering NAD(H) stimulated oligomerization of CtBP (Madison *et al.*, 2013 J. Biol. Chem, **288**, 27836-27848).

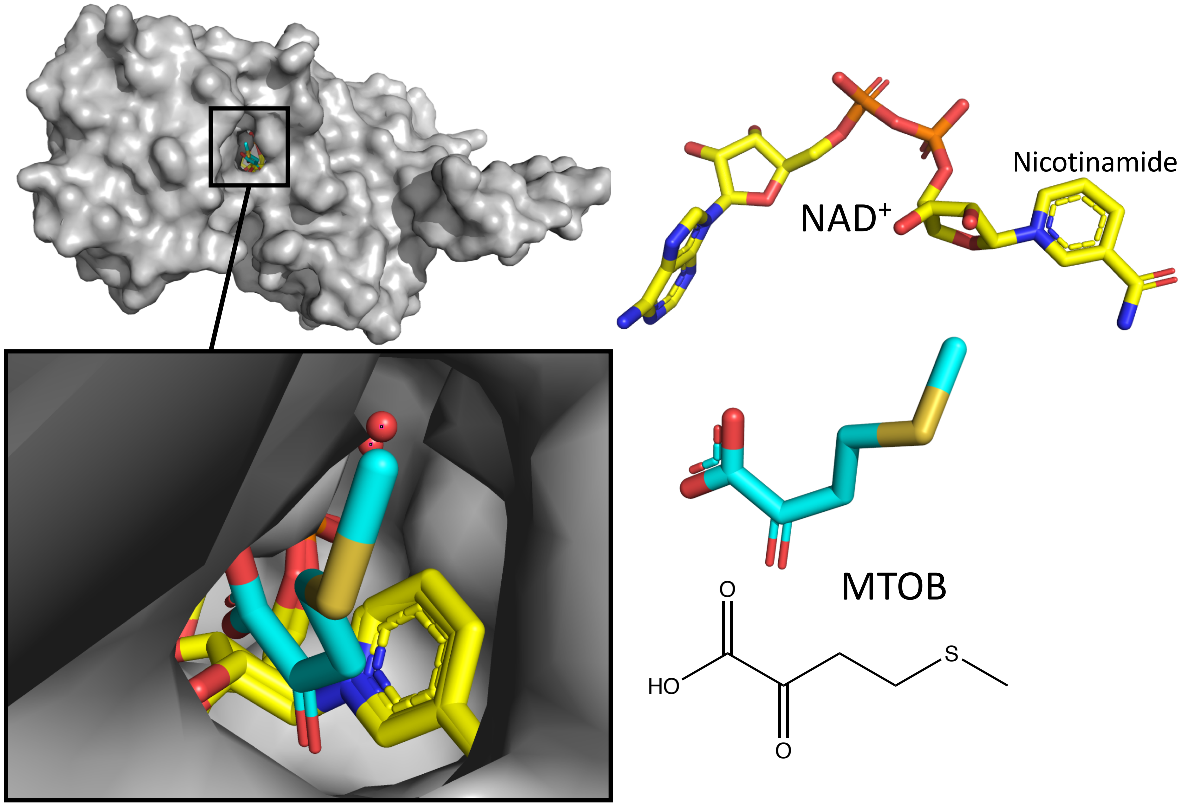

Figure S8. Binding of substrate MTOB (cyan carbon atoms) and NAD$^+$ (yellow carbon atoms) in CtBP1. The top left image shows the full subunit with a surface rendition and the small substrate cavity (box). The bottom left image includes the surface cavity with MTOB and NAD$^+$, with the six-membered nicotinamide ring apparent. (Structures of MTOB and NAD$^+$ are shown to the right.) The close proximity of the substrate and nicotinamide binding pocket suggests that compounds binding in the active site could be modified to project into the nicotinamide binding pocket to interfere with NAD(H) binding. The findings reported in this paper demonstrating that interactions involving bound nicotinamide are dispensable for CtBP activation suggests that such inhibitors could be effective in interfering with CtBP tetramer formation and transcriptional activity.

Table S1. Oligomerization of CtBP1 and CtBP2 with NAD moieties (Figure 1)

SEC Column #1	Compound	Weight-Avg Mol. Mass **M_w** (kDa)	Elution Peak Concentration (μM)	% tetramer
CtBP1 (28-440)	10μM NADH	173	3.1	72
	10μM NAD+	169	3.1	67
	1mM ADP	167	1.7	64
	1mM AMP	173	2.6	73
	2mM Adenosine	111	1.6	10
	2mM Nicotinamide	128	1.8	23
	2mM NicoRibose	123	1.8	18
	0.5mM NMN	122	1.8	17
	No NAD moiety	128	1.7	22
CtBP2 (31-445)	10μM NADH	176	2.0	73
	10μM NAD+	169	1.9	63
	1mM ADP	171	1.8	64
	1mM AMP	132	1.6	24
	2mM Adenosine	121	1.6	15
	2mM Nicotinamide	129	1.6	21
	2mM NicoRibose	129	1.6	22
	0.5mM NMN	129	1.6	22
	No NAD moiety	129	1.6	22
CtBP1 (28-375)	10μM NADH	150	2.2	75
	1mM ADP	141	1.7	60
	1mM AMP	148	1.1	71
	1mM Adenosine	97	1.7	11
	10mM Nicotinamide	96	1.8	10
	0.4mM NMN	102	1.6	15
	No added NAD moiety	103	1.8	16
CtBP2 (31-445)*	10μM NADH	184	3.2	87
	1mM ADP	178	3.2	77
	1mM AMP	125	0.8	19
	1mM Adenosine	137	1.6	27
	10mM Nicotinamide	158	3.3	49
	0.4mM NMN	140	1.5	30
	No added NAD moiety	158	3.1	48

*We suspect that the significantly lower M_w values observed with AMP, adenosine and NMN (nicotinamide mononucleotide) compared with no added NAD moiety in the SEC column #1 experiments results from competition of these compounds with residual bound NAD(H) in our purified CtBP2. (Later we were able to remove more, but not all, residual NAD(H) for these experiments.)

Table S2. AMP/ADP dependence of CtBP1/CtBP2 tetrameric assembly (Figure 2)

	Compound	Weight-Avg Mol. Mass **M**$_w$ (kDa)	Elution Peak Concentration (μM)	% tetramer
SEC Column #2				
CtBP1 (28-440)	1mM ADP	167, 170, 171	1.7, 1.7, 2.5	64, 67, 70
	100 μM ADP	159, 156, 158	1.5, 1.5, 1.5	53, 49, 52
	31.6 μM ADP	136, 134, 140	1.3, 1.3, 1.3	29, 27, 32
	10 μM ADP	125, 124, 123	1.4, 1.4, 1.2	20, 19, 18
	1 μM ADP	119, 119, 114	1.3, 1.3, 1.1	15, 15, 12
	1mM AMP	175, 177	2.6, 2.8	77, 78
	100 μM AMP	158, 158	2.0, 1.7	52, 52
	31.6 μM AMP	141, 135	1.8, 1.4	33, 28
	10 μM AMP	133, 124	1.8, 1.4	27, 19
	1 μM AMP	128, 120	1.8, 1.3	22, 16
CtBP2 (31-445)	1mM ADP	169, 170	0.6, 0.8	62, 64
	100 μM ADP	163, 164	0.8, 0.8	55, 56
	31.6 μM ADP	142, 145	0.7, 0.7	32, 35
	10 μM ADP	135, 135	0.8, 0.8	26, 26
	1 μM ADP	126, 124	0.7, 0.7	19, 17
	1mM AMP	132	1.3	24
	100 μM AMP	129	1.6	22
	31.6 μM AMP	127	1.4	19
	10 μM AMP	132	1.4	24
	1 μM AMP	129	1.3	22
SEC Column #1				
CtBP1 (28-375)	1 mM ADP	142	1.7	60
	300 μM ADP	146	1.4	67
	100 μM ADP	124	1.5	36
	10 μM ADP	105	1.6	17
	1 mM AMP	148	1.1	71
	320 μM AMP	139	1.1	56
	100 μM AMP	105	1.1	28
CtBP2 (31-445)	1 mM ADP	179	3.2	77
	300 μM ADP	173	1.6	69
	100 μM ADP	169	1.6	63
	10 μM ADP	149	1.7	39
	1 mM AMP	125	0.8	19
	320 μM AMP	124	0.8	18
	100 μM AMP	123	0.8	17

Table S3. Competition between NADH and NAD moieties (Figure 3)

	Compound	Weight-Avg Mol. Mass **M**$_w$ (kDa)	Elution Peak Concentration (μM)	% tetramer
SEC Column #3				
CtBP1 (28-440)	50nM NADH	128	1.7	22
	50nM NADH + 2mM Adenosine	119	1.6	15
	50nM NADH + 2mM Nicotinamide	127	1.6	21
	50nM NADH + 2mM Nicotinamide Ribose	124	1.7	19
CtBP2 (31-445)	50nM NADH	134	0.6	25
	50nM NADH + 2mM Adenosine	125	0.6	18
	50nM NADH + 2mM Nicotinamide	132	0.6	24
	50nM NADH + 2mM Nicotinamide Ribose	129	0.6	21
SEC Column #2				
CtBP2 (31-445)	50nM NADH	145	1.4	35
	50nM NADH + 3mM Adenosine	118	1.4	14
	50nM NADH + 5mM Nicotinamide	136	1.5	27
SEC Column #3				
CtBP1 (28-440)	50nM NADH	133	1.5	27
	50nM NADH + 2mM Adenosine	121	1.5	17
	50nM NADH + 5mM Nicotinamide	125	1.7	20
	50nM NADH + 2mM Nicotinamide Ribose	127	1.7	22
CtBP1 (28-440)	50nM NADH	141	2.7	33
	50nM NADH + 2mM Adenosine	128	2.9	22
	50nM NADH + 5mM Nicotinamide	135	2.8	26
	50nM NADH + 2mM Nicotinamide Ribose	138	2.6	31
CtBP2 (31-445)	50nM NADH	144	1.2	34
	50nM NADH + 2mM Adenosine	136	1.2	27
	50nM NADH + 2mM Nicotinamide Ribose	143	1.2	33

Table S4. MALS results on CtBP1 and CtBP2 mutants (Figure 5)

	Compound	Weight-Avg Mol. Mass $\mathbf{M_w}$ (kDa)	Elution Peak Concentration (µM)	% tetramer
Figure 5A - SEC Column #3				
CtBP1 (28-440) V185T	1mM AMP	184	2.1	91
CtBP1 (28-440) V185T	100µM AMP	172	1.9	71
CtBP1 (28-440) WT	1mM AMP	173	2.6	72
CtBP1 (28-440) WT	100µM AMP	158	2.0	52
CtBP1 (28-440) V185T Column #4)	1mM AMP	180	1.6	84
Figure 5B: SEC Column #4				
CtBP1 (28-440) L182F	1mM AMP	133	1.0	26
CtBP1 (28-440) L182F, L217V	1mM AMP	169	1.4	66
CtBP1 (28-440) WT	1mM AMP	171	1.2	70
Figure 5C: Column #4				
CtBP1 (28-440) ***11Mut*** S124A, L182F, V185T, G238N, T264A, L217V, K348R, E330A, R333T, V211I, A214S	1mM AMP	167, 167, 164	2.7, 1.4, 1.1	63, 64, 59
CtBP1 (28-440) ***11Mut*** S124A, L182F, V185T, G238N, T264A, L217V, K348R, E330A, R333T, V211I, A214S	10µM NADH	174	1.6	77
CtBP1 (28-440) ***13Mut*** S124A, L182F, V185T, G238N, T264A, L217V, K348R, E330A, R333T, V211I, A214S, V120I, A122S	1mM AMP	81, 87, 94	2.1,1.1, 2.5	0, 0, 0
CtBP1 (28-440) ***13Mut*** S124A, L182F, V185T, G238N, T264A, L217V, K348R, E330A, R333T, V211I, A214S, V120I, A122S	10µM NADH	171	1.7	70
Figure 5D: Column #4				
CtBP2 (31-445) I126V, S128A, A130S	1mM AMP	176	1.5	66
CtBP2 (31-445) I126V, S128A, A130S	10µM NADH	178	1.5	69
Minimal Dehydrogenase Domain: Column #4				
CtBP1 (28-353)	1mM AMP	128	0.6	52
CtBP2 (31-364)	1mM AMP	98	1.0	12

Table S4 continued - Related mutants and combinations (Column #4)				
CtBP1 (28-440) S124A	1mM AMP	175	1.3	75
CtBP1 (28-440) G238N	1mM AMP	166	1.4	63
CtBP1 (28-440) T264A	1mM AMP	177	2.1	79
CtBP1 (28-440) L182F/V185T	1mM AMP	169	1.6	67
CtBP1 (28-440) V185T, G238N, T264A	1mM AMP	179	2.7	82
CtBP1 (28-440) L182F, V185T, T264A	1mM AMP	171	1.6	69
CtBP1 (28-440) L182F, T264A	1mM AMP	154	0.5	47
CtBP1 (28-440) S124A, L182F, V185T, G238N, T264A, L217V	1mM AMP	179, 178	2.3, 1.1	84, 81
CtBP1 (28-440) S124A, L182F, V185T, G238N, T264A	1mM AMP	169	1.8	67
CtBP1 (28-440) V185T, T264A	1mM AMP	177	2.6	79
CtBP1 (28-440) L182F, V185T, G238N, T264A	1mM AMP	172	1.2	72
CtBP1 (28-440) V120I, A122S, S124A	1mM AMP	90	1.5	0
CtBP1 (28-440) V120I, A122S, S124A	10μM NADH	143	2.6	35
CtBP1 (28-440) V120I, A122S, S124A, K348R	1mM AMP	140	1.9	32
CtBP1 (28-440) V120I, A122S, S124A, K348R	10μM NADH	156	2.5	49
CtBP1 (28-440) V120I, A122S, S124A, V185T	1mM AMP	149	2.6	42
CtBP1 (28-440) V120I, A122S, S124A, V185T	10μM NADH	157	3.3	50
CtBP2 (31-445) T191V	1mM AMP	159	2.2	45
